# Effects of exercise therapy on disability, mobility, and quality of life in the elderly with chronic low back pain: a systematic review and meta-analysis of randomized controlled trials

**DOI:** 10.1186/s13018-023-03988-y

**Published:** 2023-07-19

**Authors:** Shi-kun Zhang, Mei-ling Gu, Ting Zhang, Hong Xu, Su-jie Mao, Wen-sheng Zhou

**Affiliations:** 1 Department of Police Physical Education, Jiangsu Police Institute, Nanjing, Jiangsu Province, China; 2 Nanjing Tian-zheng Primary School, Nanjing, China; 3 Department of Sport & Health Science, College of Natural Science, Sangmyung University, Seoul, Korea; 4 Graduate School of Nanjing University of Physical Education, Nanjing Sport Institute, Nanjing, China; 5 Department of Physical Education, Nanjing Xiao-Zhuang University, Nanjing, China

**Keywords:** Elderly, Physical activity, Exercise, Rehabilitation, Chronic low back pain

## Abstract

**Background:**

Exercise is an effective treatment in chronic low back pain (CLBP), but there are few studies on CLBP in the elderly, and the intervention effect is controversial. We aimed to compare the efficacy of different exercises therapy on CLBP, dysfunction, quality of life, and mobility in the elderly.

**Methods:**

We searched Web of Science, MEDLINE, Cochrane Library, Chinese National Knowledge Infrastructure, EMBASE, and PubMed from the database inception till December 31, 2022. The publication languages were Chinese and English. Randomized controlled trials (RCTs) of exercise intervention in the elderly (≥ 60 years) with CLBP were included. Two reviewers independently extracted the data and evaluated them using the Revised Cochrane Risk of Bias Tool for Randomized Trials 2 (RoB2). The pooled effect sizes on different aspects of outcome measures were calculated.

**Results:**

Sixteen articles (18 RCTs) were included, comprising a total of 989 participants. The quality of included studies was relatively high. Meta-analysis results indicated that exercise therapy could improve visual analog scale (VAS) (WMD = − 1.75, 95% CI − 2.59, − 0.92, *p* < 0.05), Oswestry disability index (ODI) (WMD = − 9.42, 95% CI − 15.04, − 3.79, *p* < 0,005), short-form 36-item health survey physical composite summary (SF-36PCS) (WMD = 7.07, 95% CI 1.01, 13.14, *p* < 0.05), short-form 36-item health survey mental composite summary (SF-36MCS) (WMD = 7.88, 95% CI 0.09, 15.67, *p* < 0.05), and timed up and go test (TUG) (WMD = − 0.92, 95% CI − 2.22, 0.38, *p* < 0.005).

**Conclusion:**

Exercise therapy effectively improved VAS, ODI, and SF-36 indexes in the elderly. Based on the subgroup, when designing the exercise therapy regimen, aerobics, strength, and mind–body exercise (≥ 12 weeks, ≥ 3 times/week, ≥ 60 min) should be considered carefully, to ensure the safety and effectiveness for the rehabilitation of CLBP patients. More high-quality trials are needed in future to confirm the effect of exercise on SF-36 and TUG indexes.

**Supplementary Information:**

The online version contains supplementary material available at 10.1186/s13018-023-03988-y.

## Introduction

Chronic low back pain (CLBP) is the most common health problem among the elderly [93] and one of the leading causes of dysfunction and disability [78], and pain for more than three months is considered chronic low back pain [54]. CLBP patients have pain symptoms and their daily life and walking ability are affected [1, 29, 67], not only causing imbalance and gait disturbance in the elderly [48, 66] but also posing challenges to the medical system, resulting in huge social burden and economic costs [84]. The incidence of CLBP increases with age and the degree to which CLBP interferes with the elderly correlates positively with age [55]. Compared to young people, the elderly have more severe CLBP and poor recovery ability [72]. It has been pointed out in studies that the elderly are less likely to fully recover from CLBP [70], and LBP’s adverse symptoms can only be alleviated through clinical treatment and physiotherapy. Clinical treatments such as analgesics, anti-inflammatory drugs [43], and physiotherapy including muscle relaxants, spinal manipulations, and exercise therapies are available [24]. As the population structure in developed and developing countries is transitioning toward aging [59], the proportion of the elderly in the population will further increase. According to data from the United Nations, the number of people over the age of 60 years worldwide will triple by 2050 [56]. Therefore, the number of elderly affected by CLBP will be over one billion worldwide in the coming decades [92]. Therefore, the problem of CLBP in the elderly aged 60 years and above should be addressed.

In addition to clinical therapy and physiotherapy [24], exercise therapy is one of the most common therapies for CLBP [40]. Both the American College of Physicians and European Union scholars have recommended exercise therapy as the primary means of treating CLBP [3, 11]. The low function of the muscles around the spine is an important cause of persistent pain in CLBP patients [52], and exercise can increase the strength and flexibility of lumbar vertebrae and its surrounding musculoskeletal structure and re-establish the control of lumbar vertebrae stabilizing muscles, in turn improving mobility and relieving pain [16, 69, 75]. In addition, exercise plays a positive role in all aspects such as improving self-efficacy [57], social participation, anxiety, and quality of life [58]. Evans et al. [14] and Tekur et al. [79] showed that Yoga has a significant ameliorative effect on pain and dysfunction indexes in CLBP patients. Moreover, aquatic exercises [76], aerobic exercises [86], Tai Chi [25], and pilates (David [12]) can improve the indexes related to CLBP. Similarly, a meta-analysis by some scholars Adamse et al. [2, 4, 18, 26] also demonstrated that exercise has a significant effect on improving CLBP-related indexes.

Although several studies have proved that exercise can improve CLBP, past randomized controlled trial (RCT) studies mostly focused on young or middle-aged individuals [63], and little attention has been paid to CLBP in the elderly [23]. Previous meta-analyses of exercise intervention in CLBP were not specifically aimed at the elderly, and the existing research results are not suitable for generalization to the elderly [40]. Other factors (other diseases, frequent medications, etc.) in this group can also affect the effects of exercises [82]. In a meta-analysis of the treatment for CLBP by Paeck et al. [63], 274 RCTs were included, with patients over 60 years being excluded. There are also inconsistencies in the conclusions from previous studies on exercise intervention in CLBP in the elderly. Teut et al. [80] showed that exercise does not reduce the LBP index in the elderly. In the meta-analysis by Zhang et al. [92], the effect of exercise on CLBP in the middle-aged and elderly was discussed, and the elderly group was analyzed separately in the subgroup analysis without the discussion of the indexes of quality of life and mobility.

Due to few existing studies and controversial research conclusions, clinicians have no clear evidence-based medical basis for exercise interventions in CLBP of the elderly, and there remains uncertainty regarding its effects. Based on the above considerations, in this study, we compared the effects of exercise therapy on disability, mobility, and quality of life in the elderly with CLBP. This review is expected to provide a better evidence-based basis for decision-makers involved in CLBP treatment.

## Methods

This meta-analysis involved RCTs and comparisons of different exercise modes for improving disability, mobility, and quality of life in the elderly with CLBP. The published protocol (PROSPEROCRD42023427746) was implemented according to preferred reporting items for systematic reviews and meta-analysis (PRISMA) guidelines [36].

### Literature search strategy

Following the PRISMA guidelines, the retrieval strategy was formulated [64]. Six databases were systematically searched, including the Web of Science (WOS), MEDLINE, Cochrane Library, EMBASE, China National Knowledge Internet (CNKI), and PubMed. The retrieval period was from the establishment of each database to December 31, 2022, and the languages were Chinese and English. Search themes included "low back pain," "lumbago," "physical activity," "exercise," "training," "senior," "old," "elderly," "old aged," "old age," and "randomized controlled trial." To ensure the comprehensiveness of the study, the references included in the literature and other relevant systematic reviews were searched. The literature search was performed independently by two co-authors (MLG and XH). In Table 1, the WOS database is shown as an example of the specific literature retrieval strategy. Other search strategies can be found in Additional file 1.

**Table 1:** The searching strategy for WOS

Set	Search query
#1	TOPIC: “low back pain” or lumbago or “Ache, Low Back” or “Low Backaches” or “CLBP” or “Back Pain, Low” or “Low Back Ache” or “Postural low back pain” AND DOCUMENT TYPES “Article” AND LANGUAGE “English” or “Chinese”
#2	TOPIC: “physical activity” or “exercise” or “training” or “physical therapy” AND DOCUMENT TYPES “Article” AND LANGUAGE “English” or “Chinese”
#3	TOPIC: “senior” or “older” or “elderly” or “old aged” or “older age” AND DOCUMENT TYPES “Article” AND LANGUAGE “English” or “Chinese”
#4	#1 AND #2 AND #3

### Eligibility criteria

The inclusion criteria of this study strictly adhered to the "PICOS" principle proposed by Cochrane evidence-based medicine [64], “P” stands for “participants,” “I” for “intervention,” “C” for “comparisons,” “O” for “outcomes," and "S" for "study type."

The specific inclusion criteria were as follows: (1) participants–the elderly (≥ 60 years) diagnosed with CLBP (≥ 3 months), regardless of the sex; (2) interventions–the intervention measures of the experimental group included exercise therapy, while non-exercise interventions were adopted in the control group; (3) outcomes–evaluation index including at least one of the following indexes: pain index, dysfunction index, quality of life index, and mobility index; (4) study type– RCT for exercise intervention in CLBP.

Exclusion criteria were as follows: (1) the age of the subject (< 60 years); (2) non-RCT articles; (3) outcome indexes of the study did not include the above-mentioned criteria.

### Data extraction

Data extraction was performed independently by two co-authors (MLG and XH). If there was a disagreement during the extraction process, it was resolved through negotiation or a discussion with a third co-author (WSZ). Data extraction mainly included (1) basic information about the study, including first author, publication year, and nationality; (2) information about included subjects, including sex, number, and age of subjects in the experimental and control groups; (3) design of included studies, such as the exercise mode, intervention measures (time, frequency and duration); (4) outcome indexes and outcome measurement data, including measured value and index data, etc., before and after the experiment.

### Outcome indexes

There were four outcome indexes in this study, among which the main outcome indexes included (1) pain index–visual analog scale (VAS) and (2) dysfunction index–Oswestry disability index (ODI). The secondary outcome indexes included (3) quality of life indexes–short-form 36-item health survey physical composite summary/ mental composite summary (SF-36 PCS/MCS) and (4) walking test–timed up and go test (TUG).

### Risk of bias

In this study, the Revised Cochrane Collaboration Risk of Bias Tool for Randomized Trials (RoB2) was adopted to evaluate the quality of included RCT studies [77], and there were a total of 5 evaluation indexes; each evaluation index was divided into three levels of low risk,” “some concerns,” or “high risk.” Quality evaluation was conducted independently by two co-authors (SJM and WSZ) through Review Manager, and any disagreement was resolved through discussion.

### Statistical analysis

In this study, Stata version 15.1 was used for systematic reviews and meta-analysis, and Review Manager was adopted for assessing the risk of bias in the literature. The data measured in this study are continuous variables. The outcomes were expressed as the weighted mean difference (WMD) to represent the effect index. The 95% confidence interval (CI) was calculated. Both *p*-value and *I*^*2*^ are the levels of statistical difference in the between-study heterogeneity test [31]. When *p* < 0.05, the two groups combined for statistical analysis were considered to show significant differences; when *p* > 0.05, it was considered that the two groups combined showed no statistically significant difference. In this study, the effect model of heterogeneity was determined according to the *I*^*2*^ value of heterogeneity in the Cochrane Handbook for Systematic Reviews. If there was no or small heterogeneity among studies (*I*^*2*^ ≤ 50%, *p* ≥ 0.1), the fixed-effects model was used for pooled analysis. If heterogeneity was obvious (*I*^*2*^ > 50%, *p* < 0.1), pooled analyses using the random-effects model were performed. When *I*^*2*^ > 50%, subgroup analysis was performed for the sources of heterogeneity of the main indexes (VAS, ODI), which was conducted from four aspects: exercise mode (comprehensive exercise, aerobic exercise, mind–body exercise, strength exercise, or stretching exercise), exercise cycle (< 12 weeks or ≥ 12 weeks), single exercise time (< 60 min or ≥ 60 min), and exercise frequency (1–2 times/week or ≥ 3 times/week). Sensitivity analysis was used to evaluate the stability and reliability of the results. Egger's test was used to analyze the publication bias when at least ten trials were included in the meta-analysis [30].

## Results

### Search results

**Fig. 1:**
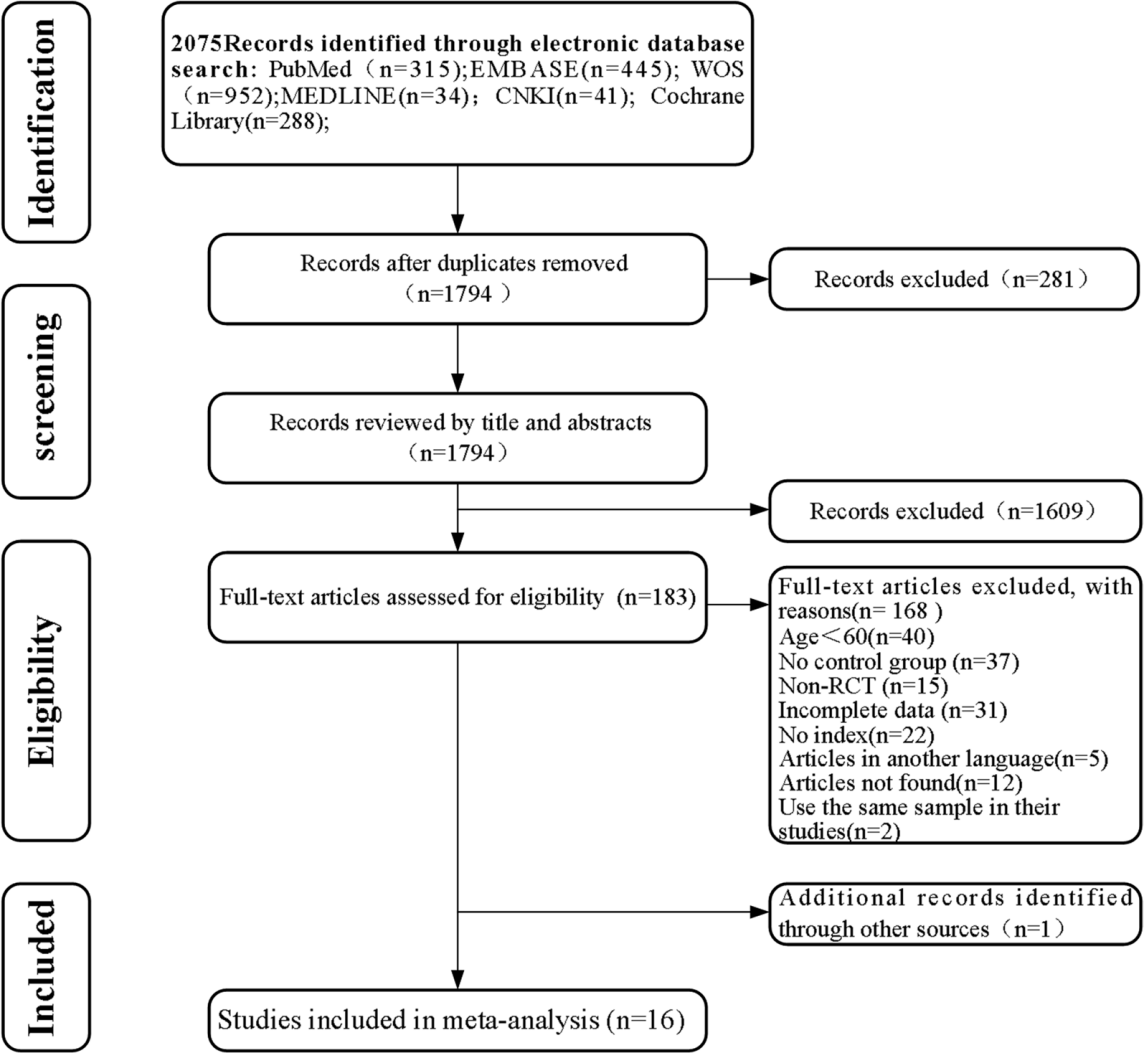
The PRISMA flowchart for included RCTs

A total of 2075 papers were obtained by searching five databases and were imported into Endnote X9 for document management; 281 duplicate papers were excluded. After reading the title and abstract, 1609 irrelevant papers were excluded. After reading the remaining 183 full-text articles, 168 were excluded due to factors such as age not meeting the inclusion criteria, lack of relevant indexes, and non-RCTs. One article was obtained from the cited references in the included literature. Finally, 16 were included for systematic reviews and meta-analysis. The literature screening process is shown in Fig. 1

### Characteristics of included studies

Sixteen papers were included, among which the experimental group described by Teut et al. [80] was divided into Qigong exercise, yoga exercise, and control groups, the experiment group of Vincent et al. [83] was divided into whole-body resistance exercise, stretching exercise and control groups. Therefore, 16 papers were finally included in this study, including 18 RCTs.

A total of 989 subjects were recruited in this study, including 496 in the experimental group and 493 in the control group. The subjects included males and females, aged ≥ 60 years. The intervention cycle was for 4–21 weeks (mostly concentrated around 12 weeks); frequency: 1–7 times/week, and duration of each intervention time: 25–90 min (mostly around 60 min). Among the 18 included studies, VAS, ODI, TUG, and SF-36 were taken as the outcome indexed in 12, 9, 3, and 5 studies, respectively. Among the included studies, the comprehensive exercise program was adopted in the experimental group of 3 studies; the strength exercise program was taken in the experimental group of 3 studies; the program of traditional physical and mental exercise was adopted in 2 studies; aerobic exercise program was taken in 5 studies, and stretch exercise program was adopted in 3 studies. No exercise intervention was adopted in the control group. Specific literature characteristics are shown in Table 2.

**Table 2:** Characteristics of included studies

Serial number	Included literature	Country	Subject characteristics	Experimental group			Control group			Outcome measures
				Sample size	Age	Exercise intervention	Sample size	Age	Intervention methods	
				(M/F)	Mean (SD)		(M/F)	Mean (SD)		
1	David Cruz-Díaz et al. [12]	Spain	Suffering from LBP for at least 3 months	53 (NR)	69.57 (2.18)	Pilates + physical therapy: 60 min × twice/week × 6 weeks	48 (NR)	72.69 (3.53)	Physical therapy	②
2	Ge et al. [19]	China	Nonspecific LBP for at least 3 months in the last year	15 (0/15)	64.6 (3.71)	Core stability training: 25–30 min × 4 times/week × 4 weeks + physiotherapy: 40 min × 4 times/week × 4 weeks	16 (0/16)	64.12 (2.96)	Physiotherapy	%1 ②
3	George et al. [20]	Greece	Lumbar spine for a period longer than 12 weeks	17 (8/9)	60–72	Treadmill training: unreported minutes × 7 times/week × 12 weeks	32 (8/24)	60–82	Written information and recommendations	②
4	Irandoust et al. [37]	Iran	With the history of back pain	18 (NR)	63.1 ± 2.4	Aquatic exercise: 60 min × 3 times/week × 16 weeks	18 (NR)	63.3 ± 1.4	ADL	③
5	Jackson et al. [38]	Canada	With chronic nonspecific LBP as diagnosed by a physician	15 (15/0)	63.0 (3.1)	Periodized resistance training: 60 min × 4 times/week × 12 weeks	15 (15/0)	57 (7.7)	ADL	①②④
6	Madadi-Shad et al. [53]	Iran	History of LBP greater than six months	18 (NR)	68.01 (2.42)	Resistance band exercise: 45–50 min × 3 times/week × 14 weeks	18 (NR)	68.9 (2.57)	ADL	①②
7	Park et al. [65]	Korea	This study specifically sought to recruit individuals with CLBP	40 (0/40)	71.5 (6.34)	Horseback riding simulator: 30 min × 3 times/week × 12 weeks	40 (0/40)	72.05 (6.82)	Watch the video	①②
8	Teut et al. [81]-A	Germany	Chronic LBP at least 6 months	61 (7/54)	73 (5.6)	Yoga: 45 min × 24 lessons × 12 weeks	57 (5/52)	72.6 (6)	No additional intervention	①④
9	Teut et al. [81]-B	Germany	Chronic LBP at least 6 months	58 (8/50)	72.4 (5.7)	Qigong: 90 min × 12 lessons × 12 weeks	57 (5/52)	72.6 (6)	No additional intervention	①④
10	Vincent et al. [83]-A	USA	Experience LBP for ≥ 6 months	17 (5/12)	68.6 (7.3)	Whole-body resistance training: unreported minutes × 3 times/week × 16 weeks	14 (5/9)	67.5 (6.4)	No exercise intervention	②
11	Vincent et al. [83]-B	USA	Experience LBP for ≥ 6 months	18 (6/12)	68.7 (7.1)	Lumbar extensor training: unreported minutes × 3 times/week × 16 weeks	14 (5/9)	67.5 (6.4)	No exercise intervention	②
12	Wang et al. [85]	China	Diagnose as CLBP by a physician	45 (NR)	66.55 (3.55)	Baduanjin: unreported minutes × 2–3 times/week × 12 weeks + magneto-caloric and interferential electrotherapy: 20 min × 3–4 times/week × 12 weeks	45	66.72 (3.21)	Magneto-caloric and interferential electrotherapy	①
13	Weiner et al. [86]	USA	Participants were community dwelling older adults with CLBP	45 (22/23)	73.9 (5.2)	Strength and flexibility and aerobic components: 60 min × twice/week × 6 weeks	44 (20/24)	73.3 (6.0)	Percutaneous electrical nerve stimulation	④
14	Yalfani et al. [88]	Iran	LBP experience over the last 6 months	13 (NR)	68 (2.94)	Virtual reality training: 30 min × 3 times/week × 8 weeks	12 (NR)	67.08 (2.9)	ADL	①④
15	Yan et al. [89]	China	Diagnose with NS-LBP for at least 3 months	10 (0/10)	68 (1.15)	24-Style Tai Chi:60 min × 3 times/week × 6 weeks	10 (0/10)	70 (1.26)	ADL	①
16	Young et al. [90]	Korea	Back pain for six months or longer	24 (NR)	Elderly	Swiss ball: 50 min × 3 times/week × 6 weeks	24 (NR)	elderly	proprioceptive neuromuscular facilitation integration pattern	%1 ③
17	Yu et al. [91]	Korea	LBP for at least 3 months	20 (0/20)	69.4 (4.1)	Waist and abdominal exercise: 40 min × 3 times/week × 8 weeks	20 (0/20)	70.4 (3.2)	Myofascial relaxation	
18	Zou et al. [94]	China	Diagnosed as CLBP by a physician	9 (NR)	65.2 (3.5)	Aquatic exercise: 60 min × 2 times/week × 21 weeks	9 (NR)	64.3 (4.8)	ADL	

①, VAS; ②, ODI; ③,TUG; ④, SF-36; M (male); F (female); NR (not report); ADL (activities of daily living).

### Risk of bias in included studies

**Fig. 2:**
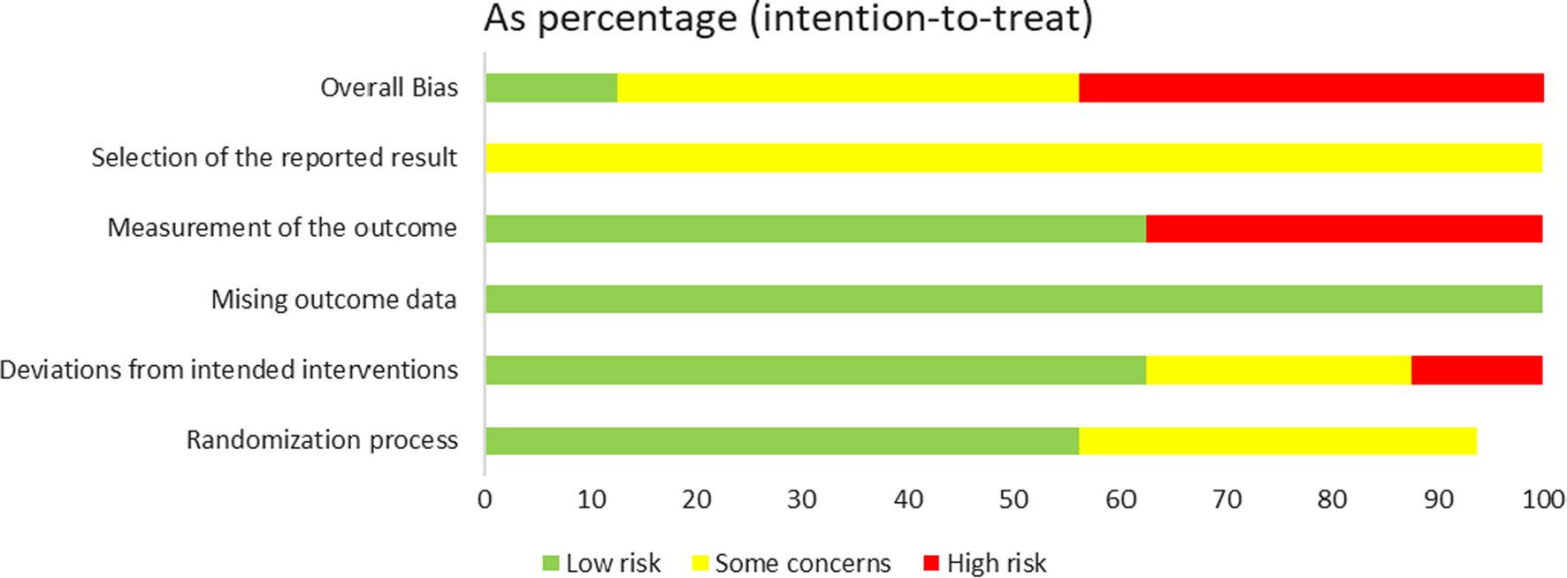
Risk of bias summary

**Fig. 3:**
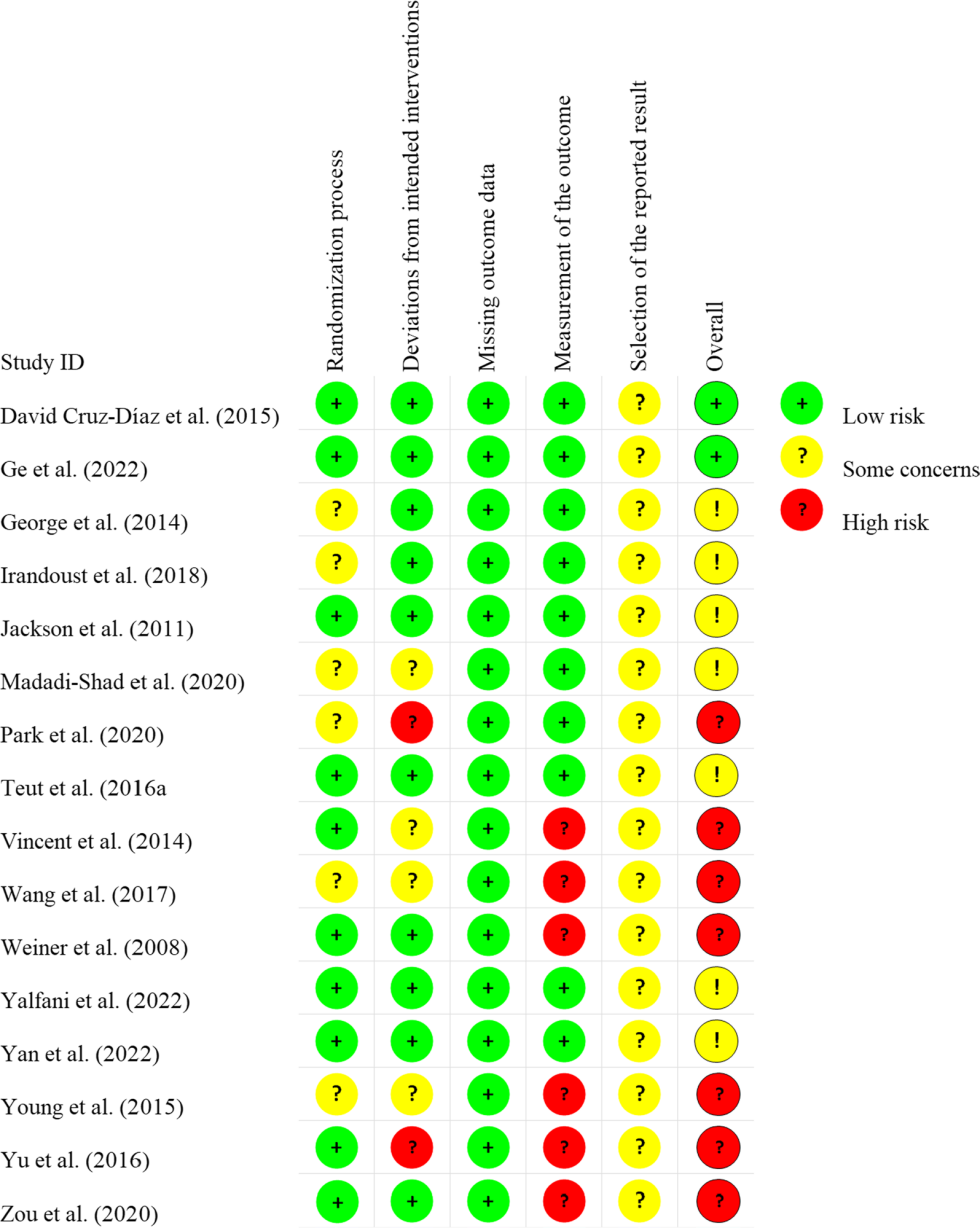
Graph depicting the risk of bias

Among 16 included papers, 7 mentioned that the overall assessment was carried out at "high risk;" 7 had “some concerns,” and 2 were at “low risk”. In the included literature, 10 were assessed as having low risk in the randomization process, while 6 showed some concerns. For deviations from intended interventions, 4 had some concerns and 2 were rated as high risk. In terms of missing outcome data, all studies were classified as high risk. For the measurement of the outcome, 6 were at low risk and 6 were at high risk. The selection of the reported result showed that 16 studies had some concerns (Figs. 2, 3).

### Effects of exercise on VAS measurements of perceived CLBP

**Fig. 4:**
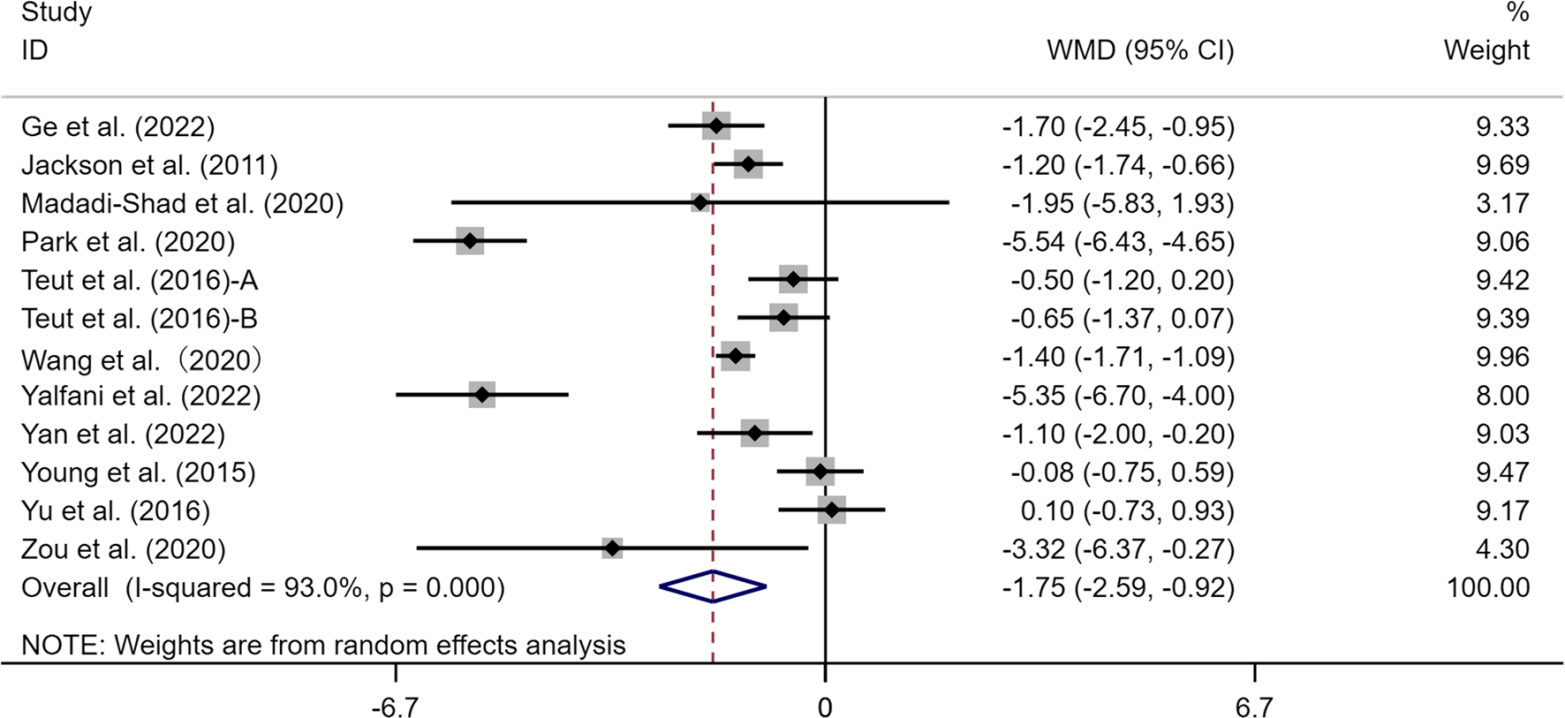
Meta-analysis of effects of different exercises on VAS measurements. *Note*: The results of heterogeneity analysis showed that *I*^*2*^ = 93.0%, *p* = 0.000

**Fig. 5:**
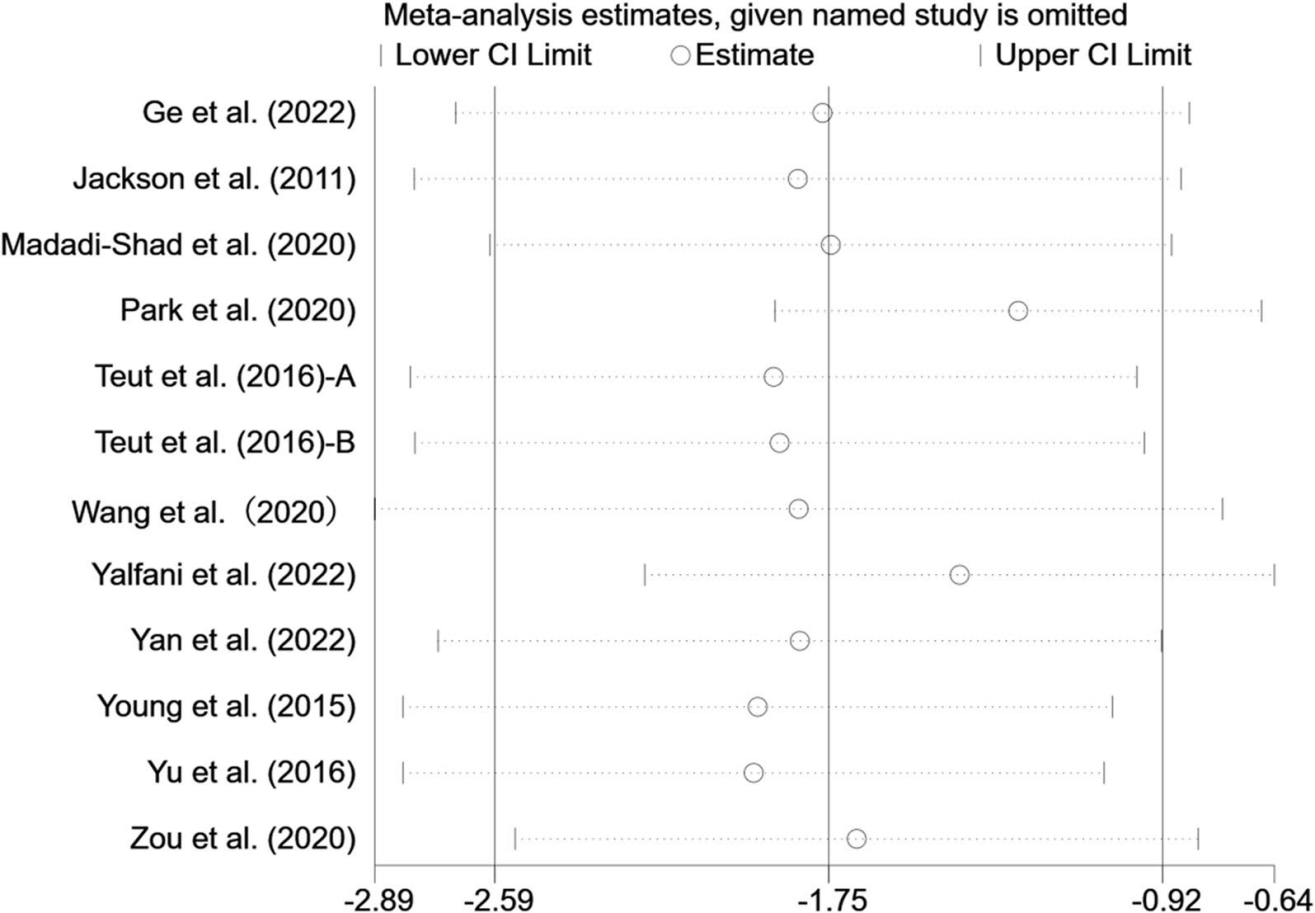
Sensitivity analysis of effects of exercise on effect size of VAS

A total of 11 articles (12 studies) provided data on the effects of exercise therapy on the VAS index in the elderly with CLBP. There was a total of 651 subjects, including 328 in the experimental group and 323 in the control group. Meta-analysis of the results from these studies showed a significant difference in the experimental group compared with the control group (WMD = − 1.75, 95% CI [− 2.59, − 0.92], *p* < 0.05). The results of the heterogeneity analysis showed that *I*^*2*^ = 93.0% (*p* < 0.05) (Fig. 4). The random-effects model was adopted for the meta-analysis. Sensitivity analysis results indicated no significant effect on the total effect size excluding any single study [39, 41] (Fig. 5).

### Effects of exercise on ODI of perceived CLBP

**Fig. 6:**
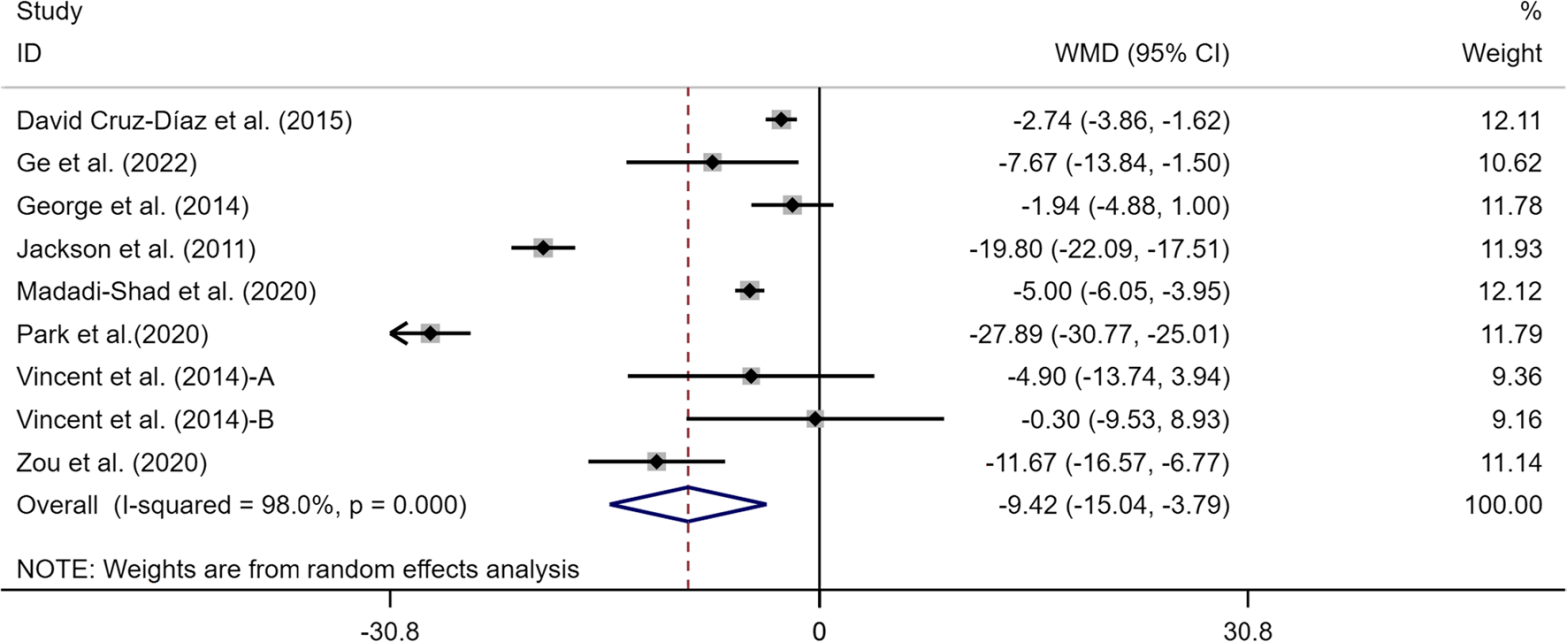
Meta-analysis of effects of different exercises on ODI measurements. *Note*: The results of heterogeneity analysis showed that *I*^*2*^ = 98.0%, *p* = 0.000

**Fig. 7:**
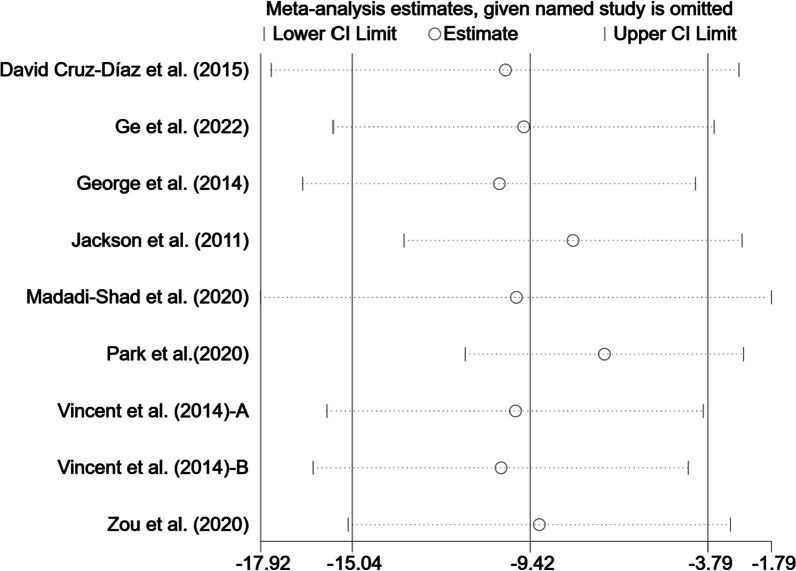
Sensitivity analysis of effects of exercise on effect size of ODI

In total, 8 articles (9 studies) involving 407 participants provided data on the ODI index. There were statistically significant associations between the exercise therapy with an improvement in ODI scores in the elderly with CLBP (WMD = − 9.42, 95% CI [− 15.04, − 3.79], *p* < 0.05, *I*^*2*^ = 98.0%) (Fig. 6). To trace the source of heterogeneity, sensitivity analysis was performed to judge the impact of each study on the effect size of ODI. As shown in Fig. 7, there was little heterogeneity among studies, and study-by-study exclusion did not greatly affect the effect size of ODI, demonstrating that the meta-analysis results are relatively stable [39, 41].

### Effects of exercise on SF-36PCS/MCS of perceived LBP

**Fig. 8:**
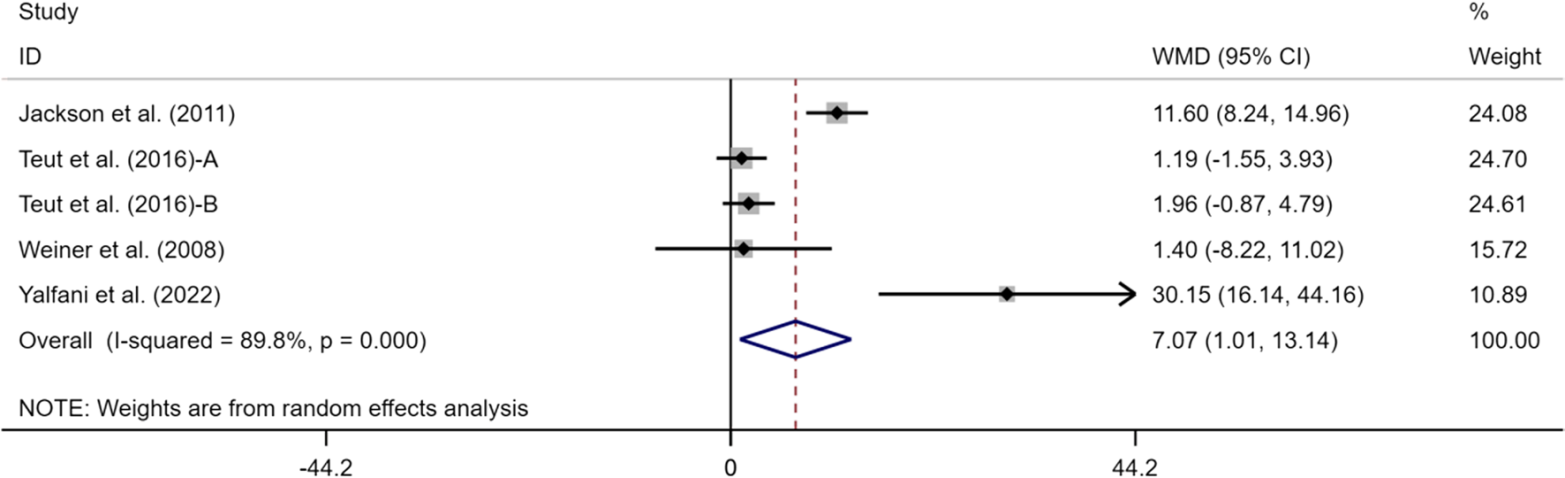
Meta-analysis of effects of different exercises on SF-36PCS measurements

**Fig. 9:**
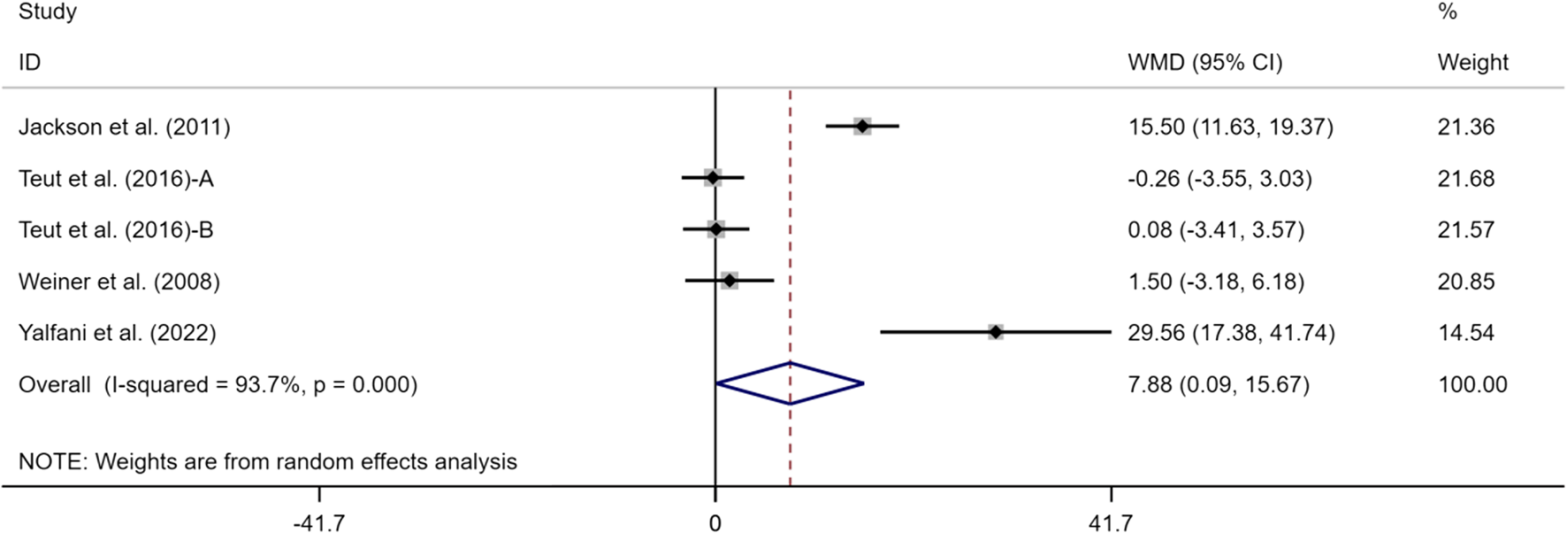
Meta-analysis of effects of different exercises on SF-36MCS measurements

Four articles (5 studies) reported SF-36PCS and SF-36MCS indexes, including 377 subjects, with 192 in the experimental group and 185 in the control group. Meta-analysis results were as follows (Figs. 8, 9): SF-36PCS: (WMD = 7.07, 95% CI [1.01, 13.14],* p* < 0.05), *I*^*2*^ = 89.8% and SF-36MCS: (WMD = 7.88, 95% CI [0.09, 15.67],* p* < 0.05),* I*^*2*^ = 93.7%. This suggests that exercise can alleviate CLBP and improve SF-36PCS and SF-36MCS indexes in the elderly in the experimental group compared with the control group, with a statistically significant difference (*p* < 0.05).

### Effects of exercise on TUG measurements of perceived LBP

**Fig. 10:**
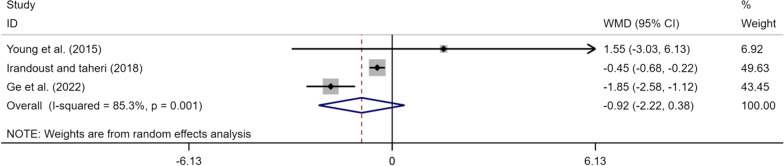
Meta-analysis of effects of different exercises on TUG measurements

Only 3 articles (3 studies) provided data on the effects of exercise therapy on the TUG index in the elderly with CLBP. A total of 115 subjects, including 57 in the experimental group and 58 in the control group were analyzed. Meta-analysis results were as follows: (Fig. 10), (WMD = − 0.92, 95% CI [− 2.22, 0.38], *p* < 0.05), *I*^*2*^ = 85.3%, suggesting that exercise tended to improve the TUG index but there was no statistically significant difference.

### Subgroup analysis for VAS and ODI

To further explore the source of heterogeneity, subgroup analysis was conducted based on the four following aspects: exercise mode, exercise cycle, single exercise time, and exercise frequency, and the findings were as follows (shown in Tables 3,4):

**Table 3:** Subgroup analysis of effects of exercise mode, exercise cycle, exercise frequency, and single exercise time on VAS measurements of perceived LBP

Subgroup type	Literature comparison group (n)	WMD (95%CI)	*P*	Heterogeneity Test	
				*I*^*2*^	*p*-value
**Exercise mode**					
Comprehensive exercise	2	− 0.7 (− 2.17, 0.76)	0.348	90.9%	0.001
Strength exercise	3	− 1.38 (− 1.81, − 0.94)	0	0	0.545
Mind–body exercise	3	− 0.7 (− 1.14, − 0.26)	0.002	0	0.58
Aerobic exercise	4	− 3.59 (− 6.98, − 0.15)	0.041	97.3%	0
**Exercise cycle**					
< 12 weeks	5	− 1.55 (− 2.99, − 0.1)	0.036	93.0%	0
≥ 12 weeks	7	− 1.95 (− 3.09, − 0.81)	0.001	93.8%	0
**Exercise frequency**					
1–2 times/week	3	− 0.71 (− 1.41, − 0.01)	0.046	35.7%	0.211
≥ 3 times	9	− 1.97 (− 3, − 0.93)	0	94.5%	0
**Single exercise time**					
< 60 min	7	− 2.06 (− 3.5, − 0.62)	0.005	96%	0
≥ 60 min	5	− 1.21 (− 1.65, − 0.76)	0	31.7%	0.21

**Table 4:** Subgroup analysis of effects of exercise mode, exercise cycle, exercise frequency, and single exercise time on ODI measurements of perceived LBP

Subgroup type	Literature comparison group(n)	WMD (95%CI)	*p*	Heterogeneity test	
				*I*^*2*^	*p*-value
**Exercise mode**					
Strength exercise	5	− 8.21 (− 13.98, − 2.43)	0.005	97.7%	0
Aerobic exercise	3	− 13.85 (− 31.2, 3.51)	0.118	98.7%	0
Stretching exercise	3	− 0.3 (− 9.53, 8.93)	0.949	-	-
**Exercise cycle**					
< 12 weeks	3	− 10.1 (− 23.0, 2.8)	0.125	98.8%	0
≥ 12 weeks	6	− 8.88 (− 17.86, 0.1)	1.94	97.9%	0
**Exercise frequency**					
1–2 times/week	2	− 6.87 (− 15.6, 1.85)	0.123	91.8%	0
≥ 3times	7	− 9.96 (− 18.15, − 1.77)	0.037	98.2%	0
**Single exercise time**					
< 60 min	4	− 16.4 (− 38.84, 6.03)	0.152	99.5%	0
≥ 60 min	5	− 10.49 (− 20.91, − 0.07)	0.048	98.3%	0

Subgroup analysis by exercise mode, aerobic exercise (WMD = − 3.59, 95% CI [− 6.98, − 0.15]), strength exercise (WMD = − 1.38, 95% CI [− 1.81, − 0.94]), and mind–body exercise (WMD = − 0.7, 95% CI [− 1.14, − 0.26]) were associated with a large reduction in VAS.

Based on the analysis of single exercise time, exercise frequency and exercise cycle (both < 60 min single time (WMD = − 2.06, 95% CI [− 3.5, − 0.62]) and ≥ 60 min (WMD = − 1.21, 95% CI [− 1.65, − 0.76]), exercise frequency (1–2 times/week, WMD = − 0.71, 95% CI [− 1.41, − 0.01]) and ≥ 3times/week (WMD = − 1.97, 95% CI [− 3.0, − 0.93]), ≥ 12 weeks exercise cycle (WMD = − 1.95, 95% CI [− 3.09, − 0.81]), and < 12 weeks (WMD = − 1.55, 95% CI [− 2.99, − 0.1]) effectively and significantly improved the VAS index.

Subgroup analysis by exercise mode suggested that only strength exercise (WMD = − 8.21, 95% CI [− 13.98, − 2.43]) was associated with a large reduction in ODI. From the analysis of single exercise time, exercise frequency (≥ 60 min single time (WMD = − 10.49, 95% CI [− 20.91, − 0.07]),  ≥ 3 times/week exercise frequency (WMD = − 9.96, 95% CI [− 8.51, − 1.77]) could effectively and significantly improve the ODI index. Exercise cycle (≥ 12 weeks exercise cycle (WMD = − 8.88, 95% CI [− 17.86, 0.1]), and < 12 weeks exercise cycle (WMD = − 10.1, 95% CI [− 23.0, 2.8]) tended to improve ODI but there was no statistically significant difference.

### Publication bias

Egger's test was adopted for assessing the publication bias in the literature associated with VAS and ODI. The study showed that when the intercept crossed the origin of the y-coordinate, the risk of publication bias was low. When the intercept did not cross the origin of the y-coordinate, publication bias existed.

**Fig. 11:**
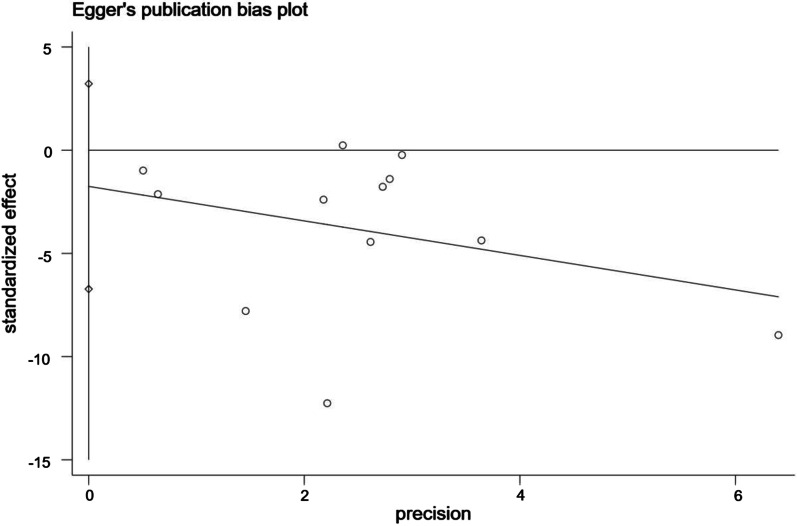
Bias analysis of the effect of exercise on VAS

**Fig. 12:**
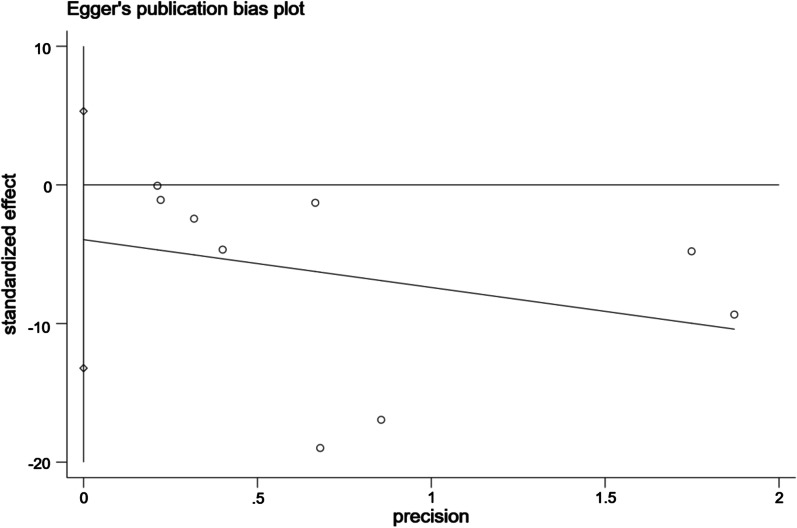
Bias analysis of the effect of exercise on ODI

As shown in Figs. 11, 12, the intervention effect of exercise on VAS indicated no publication bias (*t* = − 0.79, 95% CI − 6.72, 3.21, *p* = 0.45); the intervention effect of exercise on ODI indicated statistically significant publication bias (*t* = − 1.01, 95% CI − 13.21, 5.31, *p* = 0.594).

## Discussion

CLBP has become a global public health problem and poses a challenge [44, 60], seriously affecting the daily mobility and quality of life of the elderly [67]. However, existing exercise therapy interventions for CLBP are not suitable for the elderly [17, 40]. Therefore, this study aimed to verify the effects of different exercise modes on CLBP, dysfunction, quality of life, and mobility in the elderly. Based on the results of sixteen eligible articles, the present meta-analysis shows that exercise could significantly improve VAS, ODI, and SF-36 (SF-36PCS, SF-36MCS) indexes. However, we did not observe significant changes in the TUG index for CLBP in the elderly.

### Effects of exercise on the VAS index in elderly

Pain relief is the main index for evaluating the effects of treatment for CLBP patients [74]. In previous studies, the VAS scale was relatively often used to measure the pain symptoms of CLBP. Meta-analysis results in this study showed that exercise significantly improved the VAS index in the elderly with CLBP (WMD = − 1.75, 95% CI [− 2.59, − 0.92], *p* < 0.05). The meta-analysis of Kang and Yu [42] provides support for the results of this study (SMD = − 0.86, 95% CI [− 1.09, − 0.64], *p* < 0.05). The relatively weak strength of the trunk and abdominal muscles is an important cause of lumbago [26]. Inefficient muscle function around the spine leads to the persistence of pain in patients with CLBP[52]. Back muscle stiffness also contributes to back pain by reducing the mobility of the spine [13]. Strengthening these muscles can significantly ameliorate CLBP-related problems [49]. Andersen et al. [6] also point out that increasing muscular volume and strength may be an effective treatment strategy for pain in the elderly with CLBP. Scientific evidence suggests that the endorphins released after exercise play a physiological role in pain relief. The theory of endogenetic opioid system activation is that the release of hormones in the body after exercise can produce an analgesic effect [46]. This hormone is derived from the release of endorphins in the hypothalamus caused by exercise, which in turn relieves the response of the body to pain [17]. The study of Wong et al. [87] also supports this possible improvement mechanism and exercise can promote the secretion of *β*-endorphins, regulate the central and peripheral nervous system and reduce stress, thus achieving the effect of pain inhibition. The beneficial results in alleviating pain that we have observed lead us to recommend the use of exercise therapy (strength, mind–body, and aerobic exercise) option for the elderly affected by CLBP.

### Effects of exercise on ODI in elderly patients

Dysfunction caused by CLBP in the elderly, such as decreased activities of daily living and loss of working ability, adversely impacts patients [73]. ODI is the most commonly used scale for assessing LBP dysfunction [10, 15]. Meta-analysis results in this study showed that exercise significantly improved the ODI index in the elderly with CLBP (WMD = − 9.42, 95% CI [− 15.04, − 3.79], *p* < 0.05). The results of this study supported the latest findings of Zhang et al. [92], showing that exercise had a significant ameliorative effect on ODI (SMD = − 2.07, 95% CI [− 3.19, − 0.96], *p* < 0.05). In the subgroup analysis, both strength and aerobic exercises were found to significantly improve ODI in patients (≥ 65 years). A systematic review by Chang et al. [9] showed that strength exercise had a significant ameliorative effect on ODI for CLBP, especially waist and abdominal strength exercises. George et al. [20] put forward a different point of view based on an experiment that physical activity did not improve the ODI index in elderly patients with CLBP. The researchers believed it may be the relatively low intensity of exercise or physical activity making it difficult to produce a good exercise effect. Spinal flexibility is an important factor affecting daily life activities [7]. In functional activities, spinal flexibility may have an impact on posture control and motor performance [22]. Elderly patients with CLBP show decreased muscle strength, stability of the back muscle group, and muscle function, all resulting in reduced or lost dysfunction [21]. Hodges [32] pointed out that in the exercise intervention program for CLBP, a neuromuscular function exercise regimen should be added, with special emphasis on the exercise of pelvic and spinal muscles. Moreover, exercise can improve the control and coordination ability of trunk muscles, strengthen the stability system of lumbar vertebrae, and help restore biomechanical structures of the spine [45, 61], which in turn help improve the ability to perform physical activities and daily living functions. Improvement of the index of dysfunction also improves activity level, social and work participation, and coping strategies, and reduces fear-related beliefs regarding CLBP [27, 28], all factors that seem to be clinically significant for the elderly with CLBP.

### Effects of exercise on SF-36PCS/MCS in elderly patients

As a concise health questionnaire, SF-36 comprehensively summarizes the survival quality of the respondents from eight aspects, which can be divided into two parts: PCS and MCS [50]. Meta-analysis of this study revealed that the improvement in the quality of life index (SF-36PCS, SF-36MCS) by exercise in the elderly with CLBP showed statistical differences (SF-36PCS (WMD = 7.07, 95% CI [1.01, 13.14], *p* < 0.05), SF-36MCS (WMD = 7.88, 95% CI [0.09, 15.67], *p* < 0.05)). LBP often affects all aspects of physical and mental health in patients [35], resulting in poor quality of life [47]. Studies of the effect of exercise on the quality of life in elderly patients with CLBP are not only few but also have inconsistent conclusions. In the experimental study by Teut et al. [81], 3 months of qigong or yoga exercise intervention in the experimental group showed no ameliorative effect on the quality of life in the elderly with CLBP. The experiment of Ozkuk and Dilekci [62] suggested that aquatic exercise combined with physical therapy has an ameliorative effect on the quality of life in the elderly. The researchers believed it to be because aquatic exercise is for the whole body, and all actions in aquatic exercise affect the center of gravity of the body. Noormohammadpour et al. [61] also showed that a core stability exercise is a treatment option that can help improve the quality of life in LBP patients. The main reason for the improvement in the health and survival quality of the elderly may be related to the amelioration of pain symptoms. As a subjective feeling, pain seriously damages the physical and psychological health of the elderly, and reducing pain is conducive to the improvement in subjective well-being and quality of life [68]. Exercise may be related to the improvement in mood in the elderly. Exercise regulates the level of endorphins in the human body [34]. This substance is the "happiness hormone," which can improve people's bad moods and make them feel happy. It was long proven that exercise improves anxiety and depression and relieves stress [8], which are common psychological and emotional problems in the elderly [33]. Moreover, the improvement in the health and survival quality of the elderly may also be related to the amelioration of cognitive function. Cognition is an ability related to sensory perception and intelligence in the elderly, and exercise improves the ability to obtain, perceive, and apply external things by ameliorating the level of cognitive function, making the elderly relaxed and comfortable in dealing with daily life. This may also be an important factor in improving health degree and survival quality. An improvement in the test results of SF-36 was observed in this study.

### Effect of exercise on TUG in elderly patients

The TUG test is a comprehensive ability test mainly used to assess the motor function of patients and their risks of falling [5, 51]. The meta-analysis results revealed that the improvement in mobility index in the elderly with CLBP by exercise was statistically insignificant (WMD = − 0.92, 95% CI [− 2.22, 0.38] *p* > 0.05). A one-by-one analysis of three studies involving TUG suggested the following: Young et al. [90] revealed that after 6 weeks of exercise in proprioceptive exercise and Swiss ball exercise groups, the TUG test time between the two groups was reduced to some extent but there was no statistical difference between them (*p* > 0.05); [19] adopted therapy of core stability training combined with physiotherapy in the experimental group, and after the 4 weeks, the TUG test scores showed a significant improvement in the experimental group compared with the control group (p < 0.05). Irandoust et al. [37] showed that after 16 weeks of aquatic exercise, there was a significant improvement in the experimental group compared with the control group (*p* < 0.05). It was pointed out in previous studies that the TUG test mainly evaluates mobility, balance, walking ability, and risk of falls in the elderly. In this meta-analysis, two studies did not show a good ameliorative effect and only one study on aquatic exercise demonstrated a good effect. The number of studies included in this meta-analysis is small, resulting in an insignificant overall effect. Therefore, it is recommended to further supplement RCTs to explore the exact effect of exercise on TUG in future research.

Although a systematic review and meta-analysis of this research topic were conducted in strict accordance with the *Cochrane Handbook for Systematic Reviews of Interventions* and PRISMA principles [71], there may be some limitations and deficiencies. (1) Only English and Chinese literature were included in this study and thus the literature may not have been comprehensively included. (2) In this study, although the subgroup analysis of exercise modes was conducted, the classification of exercise types was not detailed, which may have yielded one-sided subgroup analysis results. (3) In this study, both subgroup and sensitivity analyses of sources of heterogeneity were performed but the problem of excessive heterogeneity remained unsolved, which should be addressed in future by the inclusion of high-quality literature (Additional file 2 and 3).

## Conclusion

The findings of the present meta-analysis have demonstrated that exercise therapy effectively improves pain, dysfunction, and quality of life in people over 60 years of age but there is yet no evidence that exercise can improve mobility. Based on full consideration of the cause of CLBP in the elderly, exercise therapy to improve CLBP symptoms in the elderly can focus on aerobic, strength, comprehensive, and mind–body exercises based on the exercise cycle (≥ 12 weeks), exercise frequency (≥ 3 times/week), and single exercise time (≥ 60 min). When designing the exercise therapy, the researchers should consider the aforementioned variables carefully to ensure that it would be safe and effective for the rehabilitation of CLBP patients. More high-quality trials are needed to future confirm the effect of exercise on the SF-36 and TUG indexes.

## Supplementary Information

**Additional file 1.** Literature search strategy.

**Additional file 2.** VAS ODI sensitive analysis and publication bias data.

**Additional file 3.** PRISMA Checklist.

## Data Availability

The datasets used and/or analyzed during the current study are available from the corresponding author on reasonable request.
