# Supplementary material for: Effects of exercise therapy on disability, mobility, and quality of life in the elderly with chronic low back pain: a systematic review and meta-analysis of randomized controlled trials
Source: J Orthop Surg Res. 2023 Jul 19;18:513. doi: 10.1186/s13018-023-03988-y (PMC10357808; doi:10.1186/s13018-023-03988-y)
Supplement: Supplementary file 1 — Additional file 1. Literature search strategy. [file 13018_2023_3988_MOESM1_ESM.docx]

**Appendix 1: Search Strategy**

***Database: PubMed <inception to December 31 2022>***

***Search Strategy:***

| #4 | Search: (("low back pain"[Title/Abstract] OR lumbago[Title/Abstract] OR "Ache, Low Back"[Title/Abstract] OR "Low Backaches"[Title/Abstract] OR "CLBP"[Title/Abstract] OR "Back Pain, Low"[Title/Abstract] OR "Low Back Ache"[Title/Abstract] OR "Postural low back pain"[Title/Abstract]) AND ("senior"[Title/Abstract] OR "older"[Title/Abstract] OR "elderly"[Title/Abstract] OR "old aged"[Title/Abstract] OR "older age"[Title/Abstract])) AND ("physical activity"[Title/Abstract] OR "exercise"[Title/Abstract] OR "training"[Title/Abstract] OR "physical therapy"[Title/Abstract])AND 1981/01/01:2022/12/31[Date - Publication | 315 |
| --- | --- | --- |
| #3 | Search: "physical activity"[Title/Abstract] OR "exercise"[Title/Abstract] OR "training"[Title/Abstract] OR "physical therapy"[Title/Abstract] | 927,292 |
| #2 | Search: "senior"[Title/Abstract] OR "older"[Title/Abstract] OR "elderly"[Title/Abstract] OR "old aged"[Title/Abstract] OR "older age"[Title/Abstract] | 816,244 |
| #1 | Search: "low back pain"[Title/Abstract] OR lumbago[Title/Abstract] OR "Ache, Low Back"[Title/Abstract] OR "Low Backaches"[Title/Abstract] OR "CLBP"[Title/Abstract] OR "Back Pain, Low"[Title/Abstract] OR "Low Back Ache"[Title/Abstract] OR "Postural low back pain"[Title/Abstract] | 35,786 |

***Database: EMBASE <inception to December 31 2022>***

***Search Strategy:***

| #4 | #1 AND #2 AND #3 | 445 |
| --- | --- | --- |
| #3 | 'physical activity':ti,ab,kw OR exercise:ti,ab,kw OR training:ti,ab,kw OR 'physical therapy':ti,ab,kw |  |
| #2 | 'low back pain':ti,ab,kw OR lumbago:ti,ab,kw OR 'ache, low back':ti,ab,kw OR 'low backaches':ti,ab,kw OR clbp:ti,ab,kw OR 'back pain, low':ti,ab,kw OR 'low back ache':ti,ab,kw OR 'postural low back pain':ti,ab,kw |  |
| #1 | senior:ti,ab,kw OR older:ti,ab,kw OR elderly:ti,ab,kw OR 'old aged':ti,ab,kw OR 'older age':ti,ab,kw |  |

***Database: Web of Science <inception to December 31 2022>***

***Search Strategy:***

| #4 | ((TS=( “low back pain” or lumbago or “Ache, Low Back” or “Low Backaches” or “CLBP” or “Back Pain, Low” or “Low Back Ache” or “Postural low back pain” )) AND TS=(“old” or “aged” or “senior” or “elder” or “old people” or “elderly” or “retirement” or “old aged”)) AND TS=("exercise" or "fitness" or "physical activity")AND DOCUMENT TYPES “Article” AND LANGUAGE “English” or “Chinese” AND 1981/01/01:2022/12/31[Date - Publication] | 952 |
| --- | --- | --- |
| #3 | TS=( “low back pain” or lumbago or “Ache, Low Back” or “Low Backaches” or “CLBP” or “Back Pain, Low” or “Low Back Ache” or “Postural low back pain” ) | 54227 |
| #2 | TS=("exercise" or "fitness" or "physical activity") | 787322 |
| #1 | TS=(“old” or “aged” or “senior” or “elder” or “old people” or “elderly” or “retirement” or “old aged”) | 2228306 |

***Database: MEDLINE <inception to December 31 2022>***

***Search Strategy:***

| #4 | SU ( “senior” or “older” or “elderly” or “old aged” or “older age” ) AND SU ( “physical activity” or “exercise” or “training” or “physical therapy” ) AND SU ( “low back pain” or lumbago or “Ache, Low Back” or “Low Backaches” or “CLBP” or “Back Pain, Low” or “Low Back Ache” or “Postural low back pain” )AND LANGUAGE “English” AND 1981/01/01:2022/12/31 | 34 |
| --- | --- | --- |
| #3 | SU“senior” or “older” or “elderly” or “old aged” or “older age”AND LANGUAGE “English” | 129190 |
| #2 | SU"exercise" or "fitness" or "physical activity"AND LANGUAGE “English” | 733288 |
| #1 | SU“low back pain” or lumbago or “Ache, Low Back” or “Low Backaches” or “CLBP” or “Back Pain, Low” or “Low Back Ache” or “Postural low back pain” AND LANGUAGE “English” | 52833 |

## Cochrane

#1:(“senior” or “older” or “elderly” or “old aged” or “older age”):ti,ab,kw

121395’

#2“low back pain” or lumbago or “Ache, Low Back” or “Low Backaches” or “CLBP” or “Back Pain, Low” or “Low Back Ache” or “Postural low back pain”:ti,ab,kw

13323’

#3 “physical activity” or “exercise” or “training” or “physical therapy”:ti,ab,kw

230529’

#4=#1 and #2 and #3

Total：288’

2022.1-2022.12 49”
