# Supplementary material for: Effects of exercise therapy on disability, mobility, and quality of life in the elderly with chronic low back pain: a systematic review and meta-analysis of randomized controlled trials
Source: J Orthop Surg Res. 2023 Jul 19;18:513. doi: 10.1186/s13018-023-03988-y (PMC10357808; doi:10.1186/s13018-023-03988-y)

**Appendix 2**

VAS

sensitivity analysis


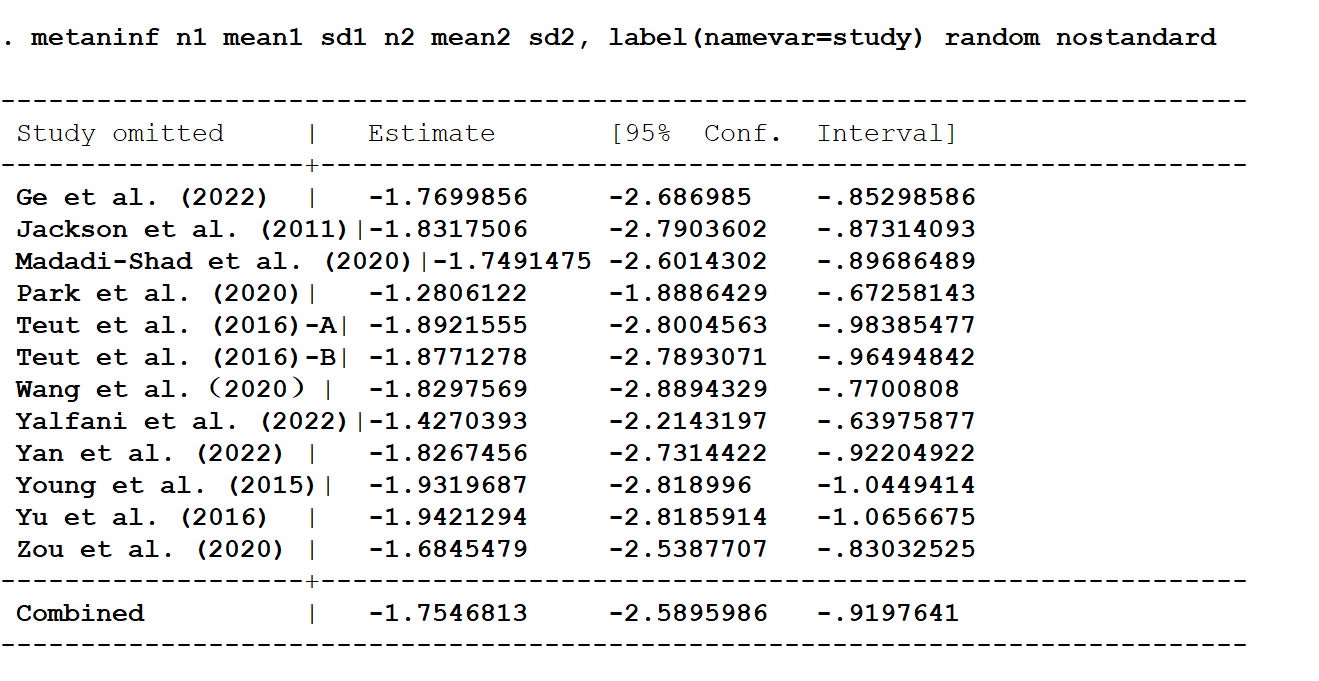


publication bias


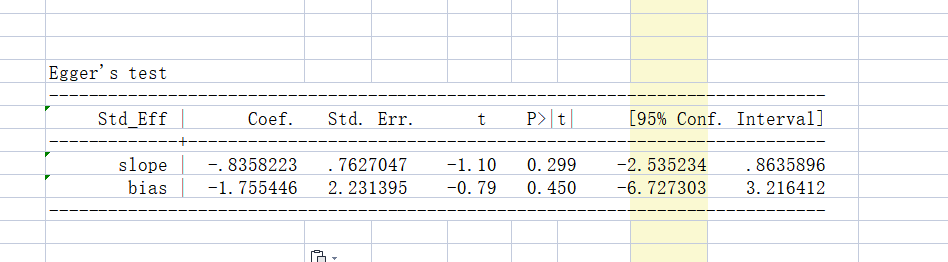


ODI

sensitivity analysis


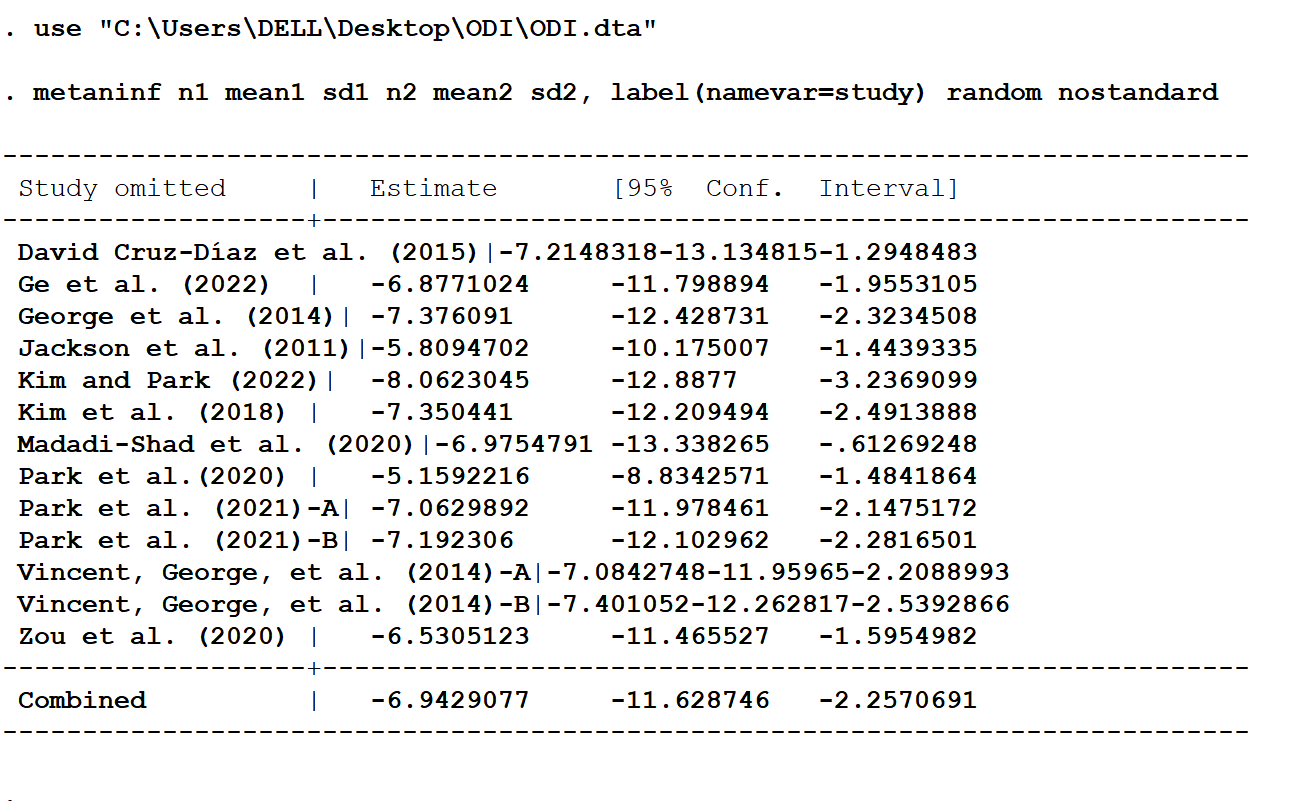


publication bias


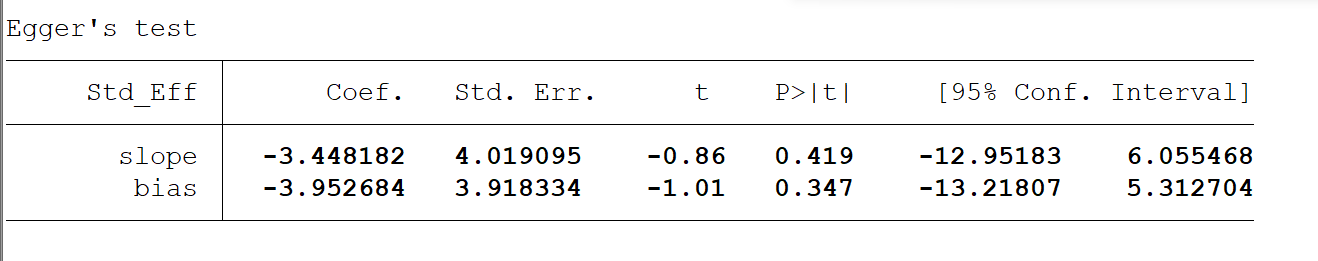

Supplement: Supplementary file 2 — Additional file 2. VAS ODI sensitive analysis and publication bias data. [file 13018_2023_3988_MOESM2_ESM.doc]
